# Compulsive gambling in ancient Indian texts

**DOI:** 10.4103/0019-5545.37674

**Published:** 2007

**Authors:** Ajit V. Bhide

**Affiliations:** Head. Department of Psychiatry, St. Marthas Hospital, Bangalore - 560094, India

The psychiatric diagnostic systems took cognizance of the syndrome of compulsive gambling as a pathological condition somewhat recently.[1,2]

Ancient Indian texts have references to the devastation that the habit of gambling can bring into one's personal, family and social life. The two outstanding examples of such references are in the Mahaabhaarata[3,4] and the Rig Veda,[5] the former in narrative and the latter as an ode to the dice that are personified.

## YUDHISHTHIRA'S FATAL FLAW

The second section of the Mahaabhaarata, the Sabha Parva, has the eldest of the heroic Paandava brothers, the otherwise virtuous Yudhisthira display a fatal flaw. The Paandavas had built a magnificent capital for themselves at Indraprastha and within it a grand palace and at its inauguration had invited their cousins the Kauravas. The eldest Kaurava, Duryodhana, first was smitten by extremely jealousy at the achievement of the Paandavas. This was aggravated by his observation of how charitable the Paandavas were being in sharing their glory and wellbeing. Further he felt humiliated when he was beguiled into some clumsiness in the hall of illusions within the palace, whereupon many including the younger Paandavas did not conceal their amusement at Duryodhana's blustering.

Burning with rage and envy he opened up to his uncle Shakuni, the prince of Gaandhaara. The scheming mind of Shakuni came up with a plan for Duryodhana to settle scores with the Paandavas.

The plan was to invite the Paandavas to the Kaurava capital of Hastinapura, ostensibly to partake their hospitality as a returned favor and then to lure Yudhishthira, known for his love of the dice, into a game against the Kaurava princes.

Despite wiser counsel from persons like the Chief Minister and uncle of both the sets of the princes, Vidura, against such an evil scheme, the plan was carried out; not only were the Paandavas invited but so were many other monarchs and princes, many famed for their love of leisure and gambling. The main event of course was between the Paandavas and their host cousins. Yudhishthira refrained from agreeing to play, enunciating the evils that gambling is fraught with and the misery it brings in its wake. He was however tricked into taking up the challenge when Shakuni hinted that the eldest Paandava was scared to play a more skilled player; and when the game began, the wily and adept Shakuni played on behalf of his nephew, Duryodhana.

One by one Yudhisthira put at stake prized jewels ornaments from his treasury, his brocades, vestments, his mineral and precious metal savings; then his livestock, horses, his armies, slaves, servants and courtesans, then his lands, his capital, his cherished palace and the crafty Shakuni with the roll of the dice kept winning them all. Protests from Vidura to Duryodhana's father, the ruling king Dhritarashtra had no effect but to evoke Duryodhana's wrath.

Desperate to win, Yudhishthira gambled away each of his four younger brothers and finally himself. The deceitful Shakuni finally suggested he put at stake his spirited and beautiful wife Draupadi and Yudhisthira fell for the ploy. Bereft of all his possessions and even his younger brothers and spouse, Yudhishthira was shorn of his pride as well for his great virtues stood him in no stead in the face of one fatal flaw: the irresistible enticement of the dice. After the pledged Draupadi was put through unprecedented humiliation before the full assembly, Dhritarashtra was woken to the cruel reality of his son's evil doings right before his eyes. He returned to Yudhishthira all that he had lost in the gambling, attempted to console Draupadi burning with rage at her shame and sent back the Paandavas to their city with honor.

It was not long before an enraged Duryodhana forced another wager on the Paandavas. When Yudhisthira lost again to Shakuni, the Paandavas and Draupadi as terms of the wager went into perilous exile for twelve years.

Yudhishthira is depicted in the epic as being lured into gambling despite knowing its dangers and ceaselessly and mindlessly increasing the stakes, forfeiting each and every one of his material and personnel assets, blinded by the hope of some chance win which never does occur. In Vidura's words are phrased the grave hazards of gambling, which we will now look at.

## THE DYUTA SUKTA: (ODE TO THE DICE)

These hazards are elaborated in prosopopoiea, in a hymn in the tenth book of the Rig Veda (Circa 4000-1500 B.C.). Here, a gambler, addressing the dice that have ruined his life, finally beseeches them to spare him:

### The Gambler:

These nuts that once tossed on tall trees in the wind

but now smartly roll over the board, how I love them!

As alluring as a draught of Soma on the mountain,

the lively dice have captured my heart.

My faithful wife never quarreled with me

or got angry; to me and my companions

she was always kind, yet I've driven her away

for the sake of the ill-fated throw of a die.

### Chorus:

His wife's mother loathes him, his wife rejects him,

he implores people's aid but nowhere finds pity.

A luckless gambler is no more good

than an aged hack to be sold on the market.

Other men make free with the wife of a man

whose money and goods the eager dice have stolen.

His father and mother and brothers all say,

“He is nothing to us. Bind him, put him in jail!”

### The Gambler:

I make a resolve that I will not go gaming.

So my friends depart and leave me behind.

But as soon as the brown nuts are rattled and thrown,

to meet them I run, like an amorous girl.

### Chorus:

To the meeting place the gambler hastens.

Shall I win? he asks himself, hoping and trembling,

But the throws of the dice ruin his hopes,

giving the highest scores to his opponent.

Dice, believe me, are barbed: they prick and they trip,

they hurt and torment and cause grievous harm.

To the gambler they are like children's gifts, sweet as honey, but

they turn on the winner in rage and destroy him.

Fifty-three strong, this band jumps playfully,

like Savitri, the god whose statutes are true.

They pay no heed to the anger of the powerful;

the king himself bows down before them.

Downward they roll, then jump in the air!

Though handless, they master those who have hands!

Unearthly coals thrown down on the board,

though cold they burn the player's heart to ashes.

Abandoned, the wife of the gambler grieves.

Grieved too, is his mother as he wanders to nowhere.

Afraid and in debt, ever greedy for money,

he steals in the night to the home of another.

He is seized by remorse when he sees his wife's lot,

beside that of another with well-ordered home.

In the morning, however, he yokes the brown steeds

and at the evening falls stupid before the cold embers.

### The Gambler to the dice:

To the mighty chieftain of your whole band,

the one who has become the king of your troop,

to him I show my ten fingers extended.

No wealth do I withhold! I speak truly!

### Chorus:

Steer clear of dice. Till well your own field.

Rejoice in your portion and value it highly.

See there, O Gambler, your cattle, your wife.

This is the counsel of the noble Savitri.

### The Gambler to the dice:

Grant us your friendship, have mercy upon us!

Do not overwhelm us with your fierce attack!

May your anger and evil intention be assuaged!

Let the brown dice proceed to ensnare another!

This description is replete with the criteria currently listed in the diagnostic systems. The ode describes how the victim becomes bereft of wealth and material assets, family, self-respect, peace of mind and social prestige. Friends and even parents disown the gambler. The device in this marvelous verse, actually is that the gambler pleads with the dice to stay away from him and spare him that insidious knell. Cunningly, at the end, the pleading one in prayer asks the dice to go find some other victim than himself!

No other account of compulsive gambling can surpass this vivid description of the havoc that the love of the dice can bring into one's life.

## References

[1] Schwartz DG (2006). Roll the bones: The History of gambling.

[2] Bhide AV (1994). Two cases of compulsive gambling and lessons from Ancient Indian texts.

[3] Ganguli KM (1896). Translation of Vyasa's Mahaabhaarata.

[4] Rajagopalachari C (1996). The Mahabharata.

[5] Singh NS (1990). HH Wilson's translation of the Rig Veda Samhita (Enlarged).

